# Results of an Exploratory Crossover Pharmacokinetic Study Evaluating a Natural Hemp Extract-Based Cosmetic Product: Comparison of Topical and Oral Routes of Administration †

**DOI:** 10.3390/ph19020231

**Published:** 2026-01-29

**Authors:** Manav Jain, Rachel Hudson, Ariel Tarrell, Danielle J. Green, Jeffrey J. Clifford, Kevin Watt, Nicole Mihalopoulos, Joseph E. Rower, Venkata Yellepeddi, Elena Y. Enioutina

**Affiliations:** 1 Pediatrics Department, Spencer Fox Eccles School of Medicine, University of Utah, Salt Lake City, UT 84108, USA; manavjain94@gmail.com (M.J.); ariel.tarrell@hsc.utah.edu (A.T.); kevin.watt@hsc.utah.edu (K.W.); nicole.mihalopoulos@hsc.utah.edu (N.M.); joseph.rower@hsc.utah.edu (J.E.R.); venkata.yellepeddi@hsc.utah.edu (V.Y.); 2 Department of Pathology, Spencer Fox Eccles School of Medicine, University of Utah, Salt Lake City, UT 84113, USA; jeff.clifford@path.utah.edu; 3 Center for Human Toxicology, College of Pharmacy, University of Utah, Salt Lake City, UT 84112, USA

**Keywords:** clinical trial, pharmacokinetics, CBD extract, cannabidiol, tetrahydrocannabinol

## Abstract

**Background:** Hemp extracts are used topically as cosmetic products and may be ingested as dietary supplements. Some users report positive carboxy delta-9-tetrahydrocannabinol (COOH-THC) urinary tests following their use. This study evaluated systemic exposure to natural hemp extract-based cosmetic (NHEC) bioactive molecules following a single dose of oral or topical application and assessed urine THC positivity. **Methods:** Twenty healthy adults (18–50 years, males and females) of a randomized, open-label, single-dose, crossover study received the NHEC orally or topically with a 15-day washout period. Plasma samples were analyzed for cannabidiol (CBD), tetrahydrocannabinol (THC), and their metabolites using a validated liquid chromatography-tandem mass spectrometry method. Pharmacokinetic parameters were calculated by non-compartmental analysis (Phoenix^®^ WinNonlin^®^ 8.4, Pharsight Inc., Chatham, NJ, USA). Urine samples were tested for COOH-THC using commercial test strips. **Results:** All analytes, except CBD and 7-hydroxy cannabidiol (7-OH-CBD), were below the limit of quantification. Oral NHEC administration resulted in a faster T_max_ (3 h vs. 24 h) and a higher AUC_0–24_ (281 vs. 19 h·ng/mL) for CBD compared to topical administration. Urine was positive for COOH-THC in 38% of participants receiving an oral dose. **Conclusions:** A single oral dose resulted in detectable plasma CBD and 7-OH-CBD, whereas topical administration produced low and frequently BLQ CBD concentrations with 7-OH-CBD and THC-related analytes not quantifiable. Urine COOH-THC tests were positive only in participants after oral use of an NHEC but not with topical use. Given the absence of THC in the product and the lack of CBD-to-THC conversion in humans, the cause of urine positivity remains unclear.

## 1. Introduction

The hemp products obtained from *Cannabis sativa* L. are popular among consumers. The hemp oil obtained from hemp seeds is enriched in omega-3 and omega-6 fatty acids, gamma-linolenic acid, proteins, essential amino acids, carbohydrates, vitamins, minerals, and other nutrients [1]. It is generally recognized as safe (GRAS) by the Food and Drug Administration (FDA) [2]. It is used for cooking and as a dietary supplement. Hemp oil is a source of “good” fats supporting the cardiovascular system. Hemp oil may also be used in skin and hair products [1].

Cannabidiol (CBD) oil, usually extracted from the hemp plant (leaves, stems, and flowers), became very popular in the past several years. The CBD oil composition is different from that of hemp oil; it typically contains CBD, other cannabinoids, polyphenols, flavonoids, and terpenes [3]. The products may be marketed as a full-spectrum product containing all bioactive components present in the plant, including tetrahydrocannabinol (THC), or products containing exclusively CBD. In the US, the growth of *Cannabis sativa* L. and the manufacture of CBD products containing less than 0.3% of delta-9 tetrahydrocannabinol (Δ9-THC) is legal and regulated by the Agricultural Act of 2014 and the Agriculture Improvement Act of 2018 [4,5]. CBD oil possesses many health benefits, including relief from anxiety, insomnia, and pain [3,6,7]. The FDA has approved a cannabidiol-containing drug, Epidiolex, shown to be effective for the treatment of seizure disorders like Lennox–Gastaut and Dravet syndromes [8]. Cannabinoids could be beneficial for treating eczema and atopic dermatitis, potentially due to their anti-inflammatory and antimicrobial properties [9]. CBD-containing oils are often used in creams, lotions, and balms due to their anti-inflammatory, antioxidant, and moisturizing properties [10]. CBD oils, intended for cosmetic products, may be unintentionally or intentionally ingested by consumers. Some consumers reported having positive urine THC tests [11].

Since general consumers may use oil-based hemp cosmetics as topical cream or as dietary supplements, this exploratory feasibility crossover study intended to characterize the PK of the concentrations of CBD, THC, and their metabolites in the plasma of healthy volunteers receiving a single topical or oral dose of a natural hemp extract-based cosmetic (NHEC) consisting of CBD and medium-chain triglycerides (MTC) derived from coconut oil. The secondary outcomes of the study were: (i) to calculate the bioavailability of the NHEC following topical or oral administrations using the published bioavailability data, (ii) to determine whether the participants who used an NHEC would have positive urine THC tests.

## 2. Results

### 2.1. General Characteristics of Participants

**Figure 1:**
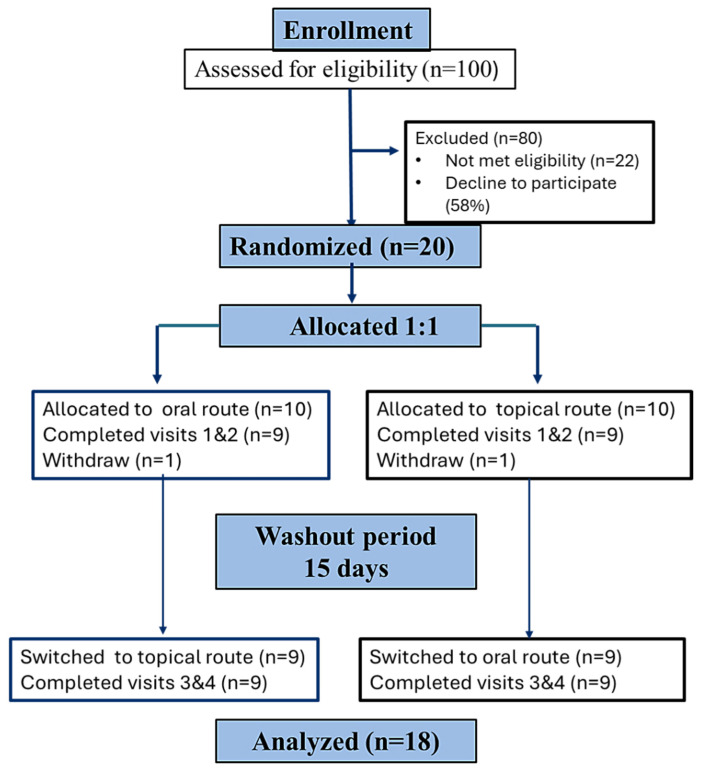
CONSORT flow diagram of participants who participated in the study, according to the CONSORT 2010 Statement.

The study coordinators approached 100 potential participants. Among them, 20 individuals met the eligibility criteria and were enrolled in the study. Among 20 enrolled participants, two decided not to participate in the second part of the study (Figure 1).

The demographic characteristics of the enrolled participants are presented in Table 1. Most of the enrolled participants were non-Hispanic white females. The average age of participants was 29.8 years. The mean BMI of the participants was 24.3. Seven out of eighteen participants were overweight with a BMI of 25.0–30.0 kg/m^2^.

### 2.2. Pharmacokinetic Analysis of CBD and 7-OH-CBD

**Figure 2:**
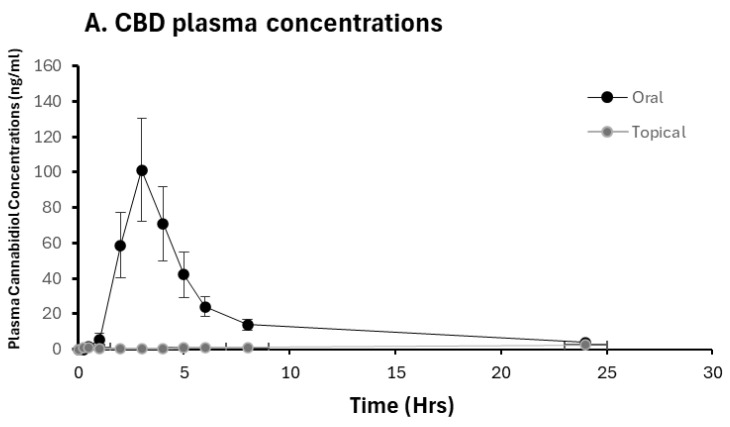

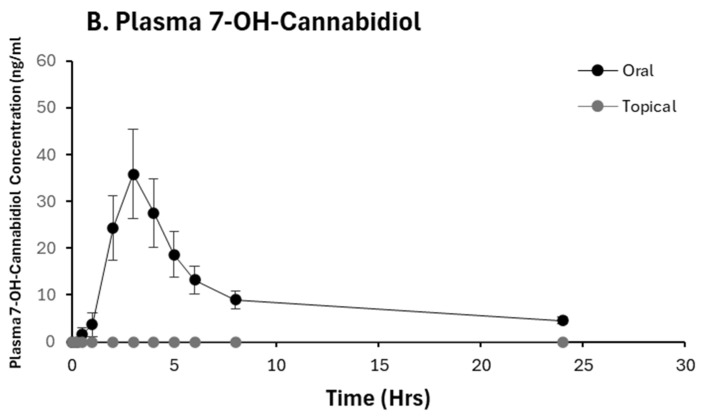
Concentration-time plot for CBD and 7OH-CBD concentrations for orally and topically administered NHEC. (**A**) Mean plasma concentrations of CBD (*n* = 20); (**B**) mean plasma concentrations of 7-OH-CBD (*n* = 18). Data presented as Mean ± SE. 7OH-CBD was not detectable following topical administration.

Only CBD and 7OH-CBD concentrations were measurable across all participant samples. The individual plasma concentrations versus time curves for each participant after administration of hemp extract are provided in Supplemental Figure S1. Pre-dose concentrations at the start of Period 2 were [below LOD/not quantifiable], supporting minimal residual carryover. For THC, THC metabolites, and cannabinol (CBN), plasma concentrations were undetectable or below the low limit of quantification (LLOQ) across samples. After topical administration, CBD was quantifiable in a subset of participants (Table 2), whereas 7-OH-CBD and other analytes were undetectable or below LLOQ, consistent with low systemic exposure from the cosmetic topical formulation. The pharmacokinetic parameters for CBD and 7-OH-CBD are presented in Table 2. The mean concentration vs. time plot at each time point of CBD and 7-OH-CBD is shown in Figure 2.

Because topical administration resulted in low and frequently below LLOQ CBD concentrations, terminal parameters (AUC_0–inf_, T_1/2_, and CL/F) could not be reliably estimated for the topical route and are therefore reported as NC (Table 2). Route comparisons were therefore focused on CBD exposure and absorption metrics supported by the data (AUC_0–24_ and T_max_; and observed C_max_ when quantifiable). Consistent with the concentration–time profiles (Figure 2), oral administration produced substantially higher systemic CBD exposure (AUC_0–24_) and an earlier T_max_ compared with topical administration.

To explore whether variability clustered by participant characteristics, we also present CBD concentration–time profiles stratified by sex and BMI range (Supplementary Figures S2 and S3). These subgroup plots are descriptive and intended to inform hypotheses for future studies rather than to support formal covariate inference.

### 2.3. CBD Bioavailability

Using previously published IV PK data of CBD and formula F = (AUC_oral/topical_ × Dose_IV_)/(AUC_IV_ × Dose_oral/topical_), the calculated absolute oral and topical bioavailability of CBD were 10.18% and 0.69%, respectively [12,13].

### 2.4. Adverse Events

There were no unexpected adverse events (AE) associated with the study product among participants. The study encountered two expected AE: vasovagal syncope with IV injection in one participant [14] and elevation of hepatic enzymes associated with NHEC (CBD) administration in another participant [15].

### 2.5. Urine Sample THC Testing

The urine of each participant was tested with the THC test 24 h after topical or oral administration of NHEC.

The urine samples were positive after oral administration of the NHEC in 38.8% of participants who completed the study (Table 3). Thirty-three percent of the urine samples were positive when NHEC was administered orally at visit 3, and one sample (5.5%) was positive when NHEC was orally administered at visit 1. None of the urine samples were positive after the topical application of the NHEC.

### 2.6. THC Test Strip Cross-Reactivity with CBD and Its Metabolites

Unexplainable positivity in the urine THC tests of some participants led us to question whether the THC test cross-reacts with CBD or its metabolites.

In vitro cross-reactivity tests determined that test strips immersed in urine samples with low CBD (50 ng/mL) and high CBD (500 ng/mL) were all negative (Supplementary Table S1). One of the replicates in lot 3 gave negative results when THC was added to the urine at 50 ng/mL (limit of detection for test strips). All other samples containing low (50 ng/mL) or high concentrations of THC (500 ng/mL) were positive. When test strips were tested in the urine containing low concentrations of THC and high concentrations of CBD, all test strips were negative.

## 3. Discussion

This exploratory feasibility crossover pharmacokinetic study characterizes the PK of CBD and 7-OH-CBD following oral and topical administrations of a CBD-based product. The analysis of the PK data obtained during the study revealed that systemic exposure from oral administration of an NHEC was substantially higher than that from topical treatment, as indicated by significantly higher C_max_ and AUC_0–24_ values. CBD (the active component of NHECs) was incorporated into MCTs, which have a high level of absorption following oral administration [16,17]. The low oral bioavailability might be due to the lipophilic nature of CBD with low solubility, incomplete absorption, and high first-pass metabolism by the liver enzymes, which might decrease bioavailability ~40-fold [18,19]. With plasma concentrations frequently falling below the lower limit of quantification, NHEC applied topically, on the other hand, showed limited systemic absorption, making it impossible to reliably estimate terminal parameters (AUC_0–inf_, T_1/2_, and CL/F) and limiting inferential comparisons to exposure metrics supported by quantifiable concentrations. Published transdermal PK data suggest that systemic exposure after topical cannabinoid delivery is often in the pg/mL to sub-ng/mL range, even with purpose-built transdermal technologies. In Varadi et al., topical application of a novel transdermal delivery system (100 mg CBD/100 mg THC) produced a CBD C_max_ of 576.52 ± 1016.18 pg/mL with large inter-individual variability [20]. The investigational product in that study incorporated formulation features designed to enhance dermal transport (e.g., penetration and dermal transport agents) [20], whereas the NHEC topical product in our study is a cosmetic formulation and was not engineered as a transdermal systemic delivery platform [20]. The frequent CBD concentration below the lower limit of quantification following topical administration may be potentially explained by the limited skin penetration of MCTs and their ability to be localized in the skin for 48 h [16].

The calculated oral bioavailability of CBD in a fasted state in our study was 10.18%. This is in line with previous research by Kolli et al., where the estimated oral bioavailability of CBD in the fasted state was 4.36–22.9% [21]. Authors also reported that the oral bioavailability of CBD followed an inverted U-shape profile at different doses. They discussed that at low doses, CBD might be completely metabolized, while at higher doses, saturation of CBD solubility became the limiting factor, resulting in lower bioavailability. In contrast, a significantly higher bioavailability of 3.5–57.3% was seen when CBD was administered in a fed state [21]. Perucca et al. reported that when the same dose of CBD was given to patients with Lennox–Gastaut syndrome and Dravet syndrome, there were large interindividual differences in the response as well as adverse events, which were partly due to low and variable oral bioavailability resulting from incomplete absorption and pre-systemic elimination [22]. Animal studies have shown low oral bioavailability of CBD (13–19%) [15]. Since bioavailability is increased following high-fat meals, lipid excipients are expected to increase oral bioavailability not only by increasing gastrointestinal absorption but also by bypassing pre-systemic elimination by directly absorbing into the lymphatic system. However, the effect on pre-systemic elimination following lipid excipients is yet to be tested.

C_max_ and AUC following oral administration showed dose dependence in previous studies [15]. However, in a single ascending dose study by Taylor, where CBD extract was administered as an oral solution via syringe, similar to our research, C_max_ increased with a trend less than dose proportionality with increasing doses for both CBD and 7-OH-CBD [19]. The dose normalized (ng/mL/mg) C_max_ for CBD and 7-OH-CBD was comparable in our study (0.26 and 0.11 at 198 mg) with Taylor’s study (CBD: 0.19 at 1500 mg, 0.18 at 3000 mg, 0.16 at 4500 mg, 0.13 at 6000 mg; 7OHCBD: 0.16 at 1500 mg, 0.11 at 3000 mg, 0.8 at 4500 mg, 0.08 at 6000 mg) [19]. A study by Crockett yielded similar results (0.25 at 750 mg) [23]. The dose-normalized C_max_ for both CBD and 7OHCBD was comparatively less when CBD extract was administered as a capsule (0.03) [24,25]. The difference could be due to different formulation use and increased sublingual absorption in oral administration in solution form. Similar findings in PK were seen when oral mucosal spray was compared with an oral capsule of CBD [23]. T_max_ did not show dose dependence in previous studies, and the T_max_ for CBD and 7-OH-CBD following oral administration in our study agreed with the earlier findings (1–4 h) [15,19,24].

In our study, the AUC_0–24_ of CBD was estimated to be 281 h·ng/mL (dose normalized—1.43 h·ng/mL/mg). In comparison, Taylor et al. reported AUC_0–48_ to be 1517, 2669, 3215, and 3696 h·ng/mL following oral CBD doses of 1500, 3000, 4500, and 6000 mg, respectively (dose normalized—1.01, 0.89, 0.71, and 0.61 h·ng/mL/mg, respectively) [19]. Similarly, Crockett et al. reported the AUC_0–96_ to be 1190 h·ng/mL following administration of 750 mg CBD (dose normalized 1.59 h·ng/mL/mg) [23]. Although comparison was limited due to differences in the study design and sampling duration, the available data suggest a trend that the dose-normalized AUC decreases with increasing dose, possibly suggesting non-linearity in CBD PK, likely due to saturable solubility.

The delayed T_max_ in the topical group suggests a slower and longer-lasting skin absorption mechanism. The poor topical bioavailability (0.69%) of the study product might indicate that the systemic availability is negligible despite this extended time to reach maximum plasma concentrations. This might be due to several reasons, such as vehicle composition, skin thickness, hydration, and permeability. The skin permeability may depend on the use of surfactants and the pH of the formulation [26]. The best skin permeation profile was reported for CBD dissolved in aqueous propylene glycol, ethanol, and Brij 98 [27]. Delivery of CBD into the skin may be enhanced by the addition of oleic acid to the formulation [28]. Capric, caprylic triglycerides, or medium-chain triglycerides were the main part of the NHEC vehicle formulation. This vehicle composition is not optimal for systemic delivery. However, NHECs are designed as cosmetic compounds, and minimal systemic bioavailability is potentially advantageous for such product use.

The NHEC contained mainly CBD and levels of THC below LLOQ. Therefore, we did not expect to find THC in the plasma or urine of participants. None of the study participants had detectable plasma levels of THC. The urine THC tests were negative in all participants receiving the NHEC topically. However, ~39% of participants receiving the NHEC orally had positive THC urine tests. The debate is still going on about whether or not CBD can be converted to THC in the human body [29]. Conversion is possible in vitro under highly acidic conditions and high temperatures but under conditions unlikely to exist in the human body. The human stomach pH is 1.5–2 [30]. Food ingestion may significantly increase stomach pH. All our participants fasted overnight and had empty stomachs in the morning. It is possible that in some of them, there were conditions that allowed conversion of CBD to THC. We also determined that the urine THC test does not cross-react with CBD and its metabolites. Additionally, our in vitro cross-reactivity test determines that high concentrations of CBD may interfere with the detection of low concentrations of THC. There were no unexpected AEs in the study participants. However, one participant had significantly elevated levels of hepatic enzymes. This is a known AE following use of or treatment with CBD products. The recent meta-analysis of the use of cannabidiol-associated hepatotoxicity concluded that cannabidiol-associated liver enzyme elevations meet the criteria of common adverse drug events [15].

In summary, this study confirmed that none of the participants had detectable levels of THC or its metabolites. Why some of the participants have had a positive urine THC test after ingestion of purified CBD products has to be further investigated.

## 4. Materials and Methods

### 4.1. Study Product

The compound used in this study is an NHEC consisting of CBD and medium-chain triglycerides (MCTs) derived from coconut oil, consisting of capric and caprylic fatty acids. The study product was provided by the Young Living Essential Oils company (YLEO, Lehi, UT, USA, sponsor of the study). The NHEC was prepared by extracting the CBD from *Cannabis sativa* whole plant. The NHEC used in this study is marketed as a topical cosmetic oil and was not designed as a transdermal systemic delivery system. The vehicle consists primarily of medium-chain triglycerides (capric/caprylic triglycerides), which may support dermal applications but are not optimized for systemic transdermal delivery. The MCTs are generally recognized as safe (GRAS) by the FDA and are highly absorbable following oral administration [16]. Based on the third-party analysis, the NHEC contains THC below the LLOQ. The shelf life of the product was 1 year.

The University of Utah Investigational Drug Services pharmacy prepared the final product. Two hundred microliters of the NHEC containing 198 mg of CBD were mixed with 0.8 mL of MCTs in a sterile syringe. For oral administration, participants were asked to swallow the study product from the syringe and take one full glass of water. When the study product was topically administered, participants were asked to apply the oil from the syringe onto the forearm.

### 4.2. Participants and Inclusion/Exclusion Criteria

Twenty healthy adult volunteers participated in the study.

*Inclusion criteria:* Healthy adult volunteers (males and females, 18–50 years old) with a body mass index (BMI) of 19–30 who did not use any recreational drug, specifically marijuana, in the past six months were included in the study.

*Exclusion criteria:* The following individuals were not enrolled in the study: (i) individuals less than 18 years of age or over 50 years of age at enrollment, (ii) individuals who previously participated in a research study involving administration of the investigational compounds within one month prior to the current study, (iii) individuals who were using any recreational drug, specifically marijuana, in the past six months, (iv) individuals with a history of alcoholism; (v) individuals who had conditions that might lead to possible interference with the study product absorption, distribution, metabolism, or excretion such as previous surgery of the gastrointestinal tract, including removal of parts of the stomach, bowel, liver, gall bladder, pancreas or adjustable gastric band surgery, impaired glucose tolerance, diabetes mellitus, renal disease, edema, or a hepatic disorder, (vi) individuals who were pregnant or could become pregnant during the study, (vii) individuals who were reporting any chronic diseases, such as mental disorders.

All eligible participants signed a consent form to participate in the trial and were randomized using randomizer.org.

### 4.3. Study Design

**Figure 3:**
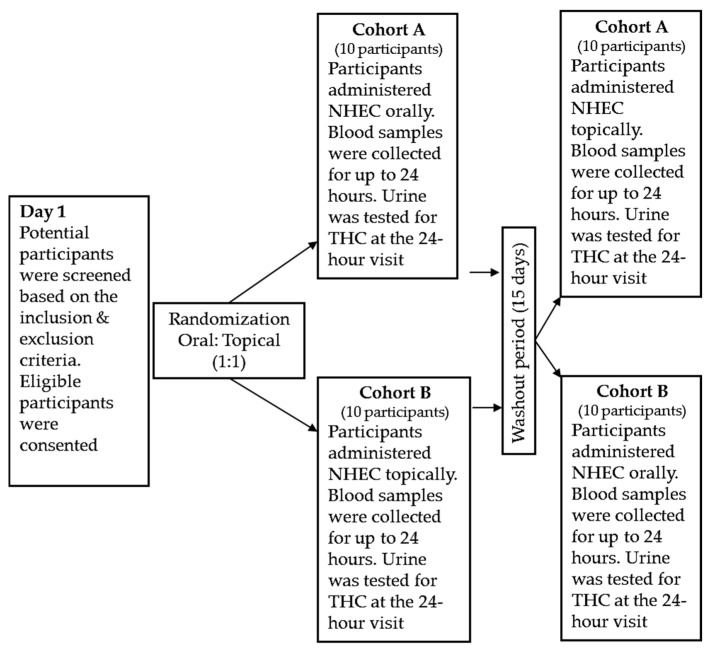
Study Design. NHEC was administered to Cohort A by oral route at visit 1, followed by a washout period, and then topically at visit 3. NHEC was administered to Cohort B by the topical route at visit 1, followed by a washout period, and then orally at visit 3.

This exploratory feasibility randomized, open-label, single-dose crossover study was conducted at the University of Utah in 2021–2024. Ethics approval was obtained from the University of Utah Institutional Review Board (IRB, No. 00139511). The registration number of the clinical trial is NCT07359976. The study design is presented in Figure 3.

This study was advertised at bulletin boards across the University of Utah campus, hospitals, and clinics. Initially, the study coordinator explained the study’s objectives to potential participants, checked their eligibility, and instructed them to abstain from food overnight the day before coming to the clinic. This study used a randomized crossover design with the two periods separated by a 15-day washout.

The office visits were scheduled with the study physicians at the Center for Clinical & Translational Science (CTSI), University of Utah. The physicians reviewed the checklist for the inclusion/exclusion criteria and collected demographic information such as age, race and ethnicity, gender, and medical history. Potential participants (if female) were asked to take a urine pregnancy test (AccuMed One Step Pregnancy Test (Houston, TX, USA), purchased at Amazon.com). If an individual met the study inclusion criteria and agreed to participate, the study physician or Principal Investigator of the study asked eligible participants to sign the IRB-approved consent form and randomly assigned them to Cohort A (oral administration) or Cohort B (topical administration). During the study, the participants were asked to refrain from using any hemp products, specifically marijuana, during the study.

*First and third office visits.* After participants were assigned to the cohorts, the CTSI personnel collected vital signs, inserted a peripheral venous catheter in the forearm’s cephalic or basilic veins, and collected a baseline blood sample according to the schedule presented in Table 4. Participants self-administered the test product orally or topically at the baseline after baseline blood samples were collected. All participants fasted overnight before dosing and did not consume food with product administration (standardized fasting conditions). Participants had breakfast 1 h and lunch 4 h after sample ingestion or application. The meals were provided by the local café and typically consisted of breakfast burritos and sandwiches for lunch. After the last blood draw, participants were instructed to return to the CTSI facility the next day at the designated time to collect the 24-h blood and urine samples.

*Second and fourth office visits.* The CTSI nurse took vital signs and collected two blood samples: a 24-h blood sample and a sample for clinical lab tests (Table 4).

**Figure 4:**
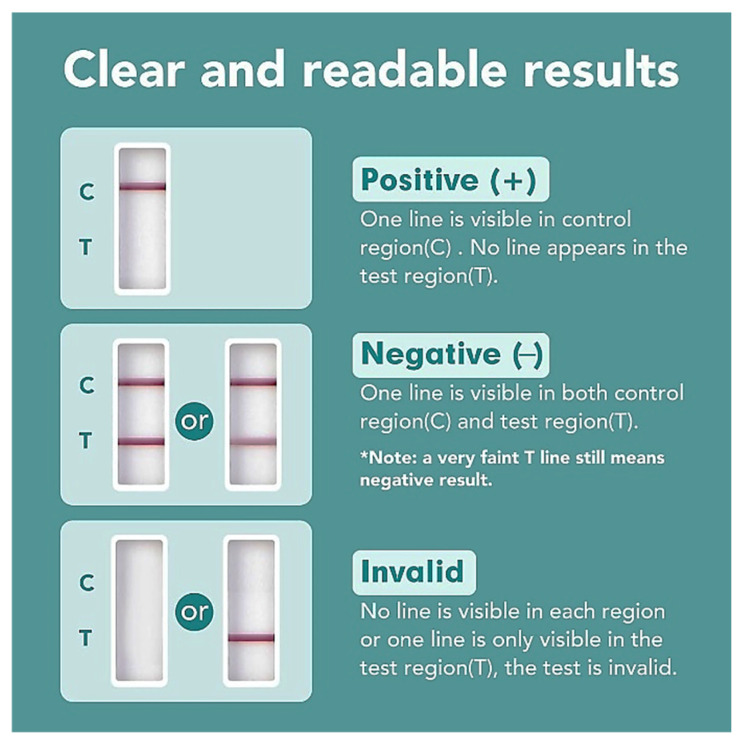
Correct reading of the Easy@Home THC Single Panel Drug Test (adopted from Amazon.com).

Twenty-four-hour collected urine samples were immediately tested for THC positivity using the Easy@Home Marijuana (THC) Single Panel Drug Test Kit (Easy Healthcare Corporation, Burr Ridge, IL, USA, THC test, purchased at Amazon.com). This test is cleared by the FDA 510(k) for over-the-counter home use and CLIA waived for professional use. The test detects THC at the standard cut-off level of 50 ng/mL within 2 h to 5 days and uses COOH-THC as a calibrator. The urine sample THC testing was performed according to the manufacturer’s recommendations (Figure 4). Urine samples were discarded after testing.

Collected blood samples were processed at the CSTI facility, and plasma samples were labeled with the code that included the de-identifying participant’s code, visit number, and time of sample collection. De-identified samples were stored at the secured CTSI site at −80 °C until use. Further sample and data analyses were performed with the de-identified samples.

### 4.4. Sample Size Rationale

This was an exploratory, within-subject crossover feasibility study intended to characterize systemic exposure and variability following oral versus topical application rather than to support confirmatory hypothesis testing. A target enrollment of 20 participants was selected to yield at least 12 evaluable completers after accounting for anticipated attrition consistent with common practice and regulatory expectations for crossover PK/BA studies that recommend a minimum of 12 evaluable subjects [31,32,33]. Eighteen participants completed both periods, providing sufficient data to summarize PK parameters and conduct paired within-subject comparisons for exposure metrics supported by quantifiable concentrations.

### 4.5. Study Outcomes

The primary outcome of this study was to determine the presence and concentrations of CBD and THC and their metabolites in the plasma of participants at various time points after oral or topical use of the product, followed by non-compartmental pharmacokinetic analysis.

The second outcome was to determine whether the urine samples of participants were positive for THC using Easy@Home test strips.

### 4.6. Plasma Analysis for CDB, THC, and Their Metabolites

Collected plasma samples were analyzed using a validated liquid chromatography-tandem mass spectrometry (LC-MS/MS) assay to measure Δ9-THC, Δ8-THC, 11-OH-THC, COOH-THC, CBD, 7-hydroxy cannabidiol (7-OH-CBD), and cannabinol (CBN) concentrations [34]. All reference standards and internal standards were purchased from Cerilliant as either 1.0 or 0.1 mg/mL solutions. The LC-MS/MS consisted of a Thermo Scientific TSQ Vantage triple quadrupole mass spectrometer interfaced with a Thermo Scientific Accela LC and autosampler (Thermo Scientific, Waltham, MA, USA). Chromatographic separation utilized a Phenomenex Luna Omega PS C18 (Phenomenex, Torrance, CA, USA) (2.1 × 100 mm, 3.0 μm particle size) analytical column with a Security Guard ULTRA C18 (Phenomenex, Torrance, CA, USA) guard column heated to a temperature of 40 °C. Mobile phase consisted of (A) 1 mM ammonium formate (pH 3.5) and (B) methanol initiated at 72%A:28%B flowing at 0.250 mL/min. Initial mobile phase conditions were held for 2.25 min, and with increased linearly to 85%B at 10.5 min, then immediately switched to 100%B, where it was held for 1.5 min before being re-equilibrated at initial conditions. Selective reaction monitoring for the analytes and their respective deuterated internal standards was utilized for quantitation. Peak area ratios of analyte to internal standard for each sample were compared to a 1/x^2^ linearly regressed calibration curve between 0.5 and 100 ng/mL (2.5 to 500 ng/mL for COOH-THC) to determine the sample concentration. The lower LLOQ for CBD and 7-OH-CBD was 0.5 ng/mL.

A 200 μL plasma aliquot was extracted for all blanks, calibrators, quality control, and participant samples in polypropylene test tubes. A 50 µL volume of internal standard was added to all tubes except double blanks. The pH was adjusted by adding 500 µL of 2 mM ammonium formate (pH 3.5) to each tube, and all tubes were mixed for 30 s on a multi-tube vortexer. A 2 mL volume of 5:1, hexane:Methyl Tertiary-Butyl Ether was added to all tubes as the extraction solvent. The tubes were capped, vortex mixed for 5 min, centrifuged at 1200× *g* for 15 min, and placed in a −80 °C freezer for 30 min. The organic upper layer was decanted into fresh polypropylene tubes and evaporated in a Turbovap (Zymark, Marshall Scientific, Hampton, NH, USA) under ~15 psi of air from a house compressed air system for ~18 min in a 40 °C water bath. The dried extracts were reconstituted in 200 µL of 50% methanol:50% water. Following a 2-min centrifugation at 1200× *g* and then a 30-s vortex mix, the reconstituted extracts were transferred to conical-bottom polypropylene autosampler vials for injection onto the LC-MS/MS system.

### 4.7. Pharmacokinetic Analysis

Pharmacokinetic parameters were determined by non-compartmental analysis using Phoenix^®^ WinNonlin^®^ software version 8.4 (Certara, Princeton, NJ, USA). Following administration of both oral and topical formulations, pharmacokinetic parameters evaluated were the maximum plasma concentration (C_max_), time to reach maximum plasma concentration (T_max_), area under the plasma concentration-time curve from zero to last measured concentration (AUC_0–24_), AUC from time zero to infinity (AUC_0-inf_), terminal (elimination) half-life (T_1/2_), and apparent clearance (CL/F). AUC was estimated using the linear up-log-down method for CBD and 7OH-CBD. T_max_ was represented by median (range), while C_max_, T_1/2_, Cl/F, and AUC were represented in geometric mean (95% confidence interval). For the calculation of AUC_0–24_ and AUC_0–Inf_, a concentration of zero is assigned to each sample at time = 0 (pre-dose). The differences in pharmacokinetic parameters after oral and topical administration were analyzed by the Wilcoxon signed-rank test using R software version 4.3.2. Because the study used a within-subject crossover design, route comparisons were paired within participants. Given the small sample size and the skewed distribution of PK parameters (with outliers and below LLOQ-related missingness), we selected the Wilcoxon signed-rank test as a nonparametric paired method.

Given the sampling schedule (frequent sampling through 8 h with a single 24-h sample), primary PK summaries emphasize C_max_ and AUC to the last measurable timepoint (AUC0–tlast) and/or AUC_0–24_, which are directly supported by observed data. Parameters requiring characterization of the terminal log-linear phase (λz), including t½ and AUC_0–inf_, were treated as secondary and interpreted cautiously, as these estimates can be unstable when the terminal phase is not well captured.

The absolute bioavailability (fraction of administered dose that reaches systemic circulation when compared with intravenous IV formulation) of CBD was also calculated following oral and topical administration, using previously published CBD PK data following IV administration [12,13]. Because IV and non-IV data were not obtained within the same protocol, the resulting bioavailability values are intended as an approximate context for systemic exposure rather than definitive absolute bioavailability estimates.

Handling of BLQ and parameter computability: Concentrations below LLOQ were treated as missing for noncompartmental calculations except that the pre-dose sample (baseline) was set to zero. PK parameters were summarized only when sufficient quantifiable post-dose concentrations were available to support the calculation. In particular, parameters requiring characterization of the terminal elimination phase (AUC_0–inf_, T_1/2_, and CL/F) were not estimated when the terminal phase could not be reliably defined. Route comparisons were restricted to CBD exposure metrics that could be supported by the observed data (AUC_0–24_ and T_max_, and observed C_max_ when quantifiable), and paired statistical testing was performed only for participants with quantifiable post-dose CBD concentrations in both crossover periods. Parameters reported as NC were not included in inferential comparisons.

### 4.8. In Vitro Analysis of Cross-Reactivity of Cannabinoid Urine Test Strips with CBD and Its Metabolites

The potential cross-reactivity of the Easy@Home Marijuana (THC) Single Panel Drug Test Kit with CBD and its metabolites was tested at the Center for Human Toxicology. Analyte-free urine was purchased from BioIVT (Woodbury, NY, USA) and prescreened using an adaptation of the previously described LC-MS/MS method [34] to demonstrate the absence of cannabinoids. Three unique urine lots were used for the experiment. Six test groups were prepared in each urine lot, containing either no CBD or Δ9-THC (negative control); low THC (50 ng/mL Δ9-THC, 11-OH-THC, and COOH-THC); high THC (500 ng/mL Δ9-THC, 11-OH-THC, and COOH-THC); low CBD (50 ng/mL CBD, 7-OH-CBD, and COOH-CBD), high CBD (500 ng/mL CBD, 7-OH-CBD, and COOH-CBD); and a final group with low THC/high CBD (50 ng/mL Δ9-THC, 11-OH-THC, and COOH-THC with 500 ng/mL CBD, 7-OH-CBD, and COOH-CBD). Samples were vortex mixed well before testing according to the manufacturer’s recommendations. Recommendations included dipping the test strip into the urine sample for 10 s, followed by a 5-min test development. The test was read as presented in Figure 2.

## 5. Conclusions

The NHEC had low bioavailability following a single oral or topical administration. Only CBD and 7-OH-CBD were detected in the plasma of participants who ingested an NHEC, and CBD was detected in the plasma of participants who topically applied the study product. No THC or its metabolites were detected in the plasma of study participants, while 38.8% of them who were taking the NHEC orally had positive THC urine tests. The potential mechanisms of the observed phenomenon need to be investigated. The liver enzymes can be elevated in some people using CBD products; therefore, participants with altered liver functions need to be closely monitored due to the potential hepatotoxicity of the CBD. Fifty-eight percent of the approached volunteers did not respond after initial contact or decided not to participate due to a conflict with their work schedule. The future CBD studies may encounter problems with recruiting participants due to the popularity of CBD supplements and cosmetic products, which are actively used by general consumers now.

## 6. Limitations

A limitation of the study is that we did not test the urine of the participants for THC before topical or oral administration of the study product. The positive tests detected after oral administration may suggest that the positive tests were due to some physiological properties of the gastrointestinal tract of the participants and potentially higher bioavailability of the oral CBD. For the topical product, the observed T_max_ occurred near the end of the intensive sampling window, and thus, the true T_max_ could occur later. In addition, because late-phase sampling was limited, parameters T_½_ and AUC_0–inf_ may be less reliable and should be interpreted cautiously; exposure comparisons are therefore best supported by AUC metrics based primarily on observed concentrations (e.g., AUC_0–tlast_/AUC0–24). A further limitation is that absolute bioavailability was estimated using historical IV CBD PK data rather than an IV arm conducted within the same study. Inter-study differences in population characteristics, formulation/excipient systems, sampling duration, and bioanalytical methods can introduce variability in AUC and therefore affect the accuracy of the absolute bioavailability estimates. Accordingly, these values should be interpreted as approximate and hypothesis-generating.

## Figures and Tables

**Table 1:** Characteristics of participants who completed the study.

Characteristics	Number (%)
**Ethnicity/race**	
Non-Hispanic, White	13 (72.2%)
Hispanic, White	1 (5.6%)
Non-Hispanic, Black	2 (11.1%)
Asian	2 (11.1%)
**Gender**	
Females	13 (72.2%)
**Age**, mean ± SD	29.8 ± 6.6
**BMI**, mean ± SD	24.3 ± 3.2

**Table 2:** Pharmacokinetic parameters of CBD and 7-OH-CBD following oral and topical NHEC administration.

Pharmacokinetic Parameter	CBD		7-OH-CBD	
	Oral	Topical	Oral	Topical
C_max_ (ng/mL)	52.13 (3.31–820.30)	NC ^$^	22.06 (2.17–224.27)	NC ^$^
T_max_ (h)	3.00 (2.00–8.00)	24.00 (0.25–24.37)	3.00 (2.00–24.00)	NC ^$^
T_1/2_ * (h)	7.9 (3.93–29.70)	NC ^$^	13.17 (6.24 -72.73)	NC ^$^
AUC_0–24_/(h·ng/mL)	281 (35.45–2226.58)	19 (2.42–149.14)	166.77 (26.72–1040.96)	NC ^$^
AUC_0–inf_/(h·ng/mL)	342.30 (225.64–510.27)	NC ^$^	275.65 (51.60–1472.57)	NC ^$^
Cl/F (L/h)	0.57 (0.09–3.52)	NC ^$^	0.72 (0.13–3.84)	NC ^$^

Pharmacokinetic analysis was conducted using CBD and 7-OH-CBD plasma concentrations. Data presented as Geometric mean (95% geometric CI); * Data presented as median (range), ^$^ NC, not computed due to insufficient quantifiable concentrations (e.g., values frequently BLQ/not detected) to support reliable estimation; NC parameters were not used for route-comparison statistics.

**Table 3:** Urine sample THC-positivity after oral administration of NHEC.

Visit 1, *n* (%) *	Visit 3, *n* (%) *	Total THC Positivity, *n* (%) *
1 (5.5%)	6 (33.3%)	7 (38.8%)

* Percent of participants who completed the study (*n* = 18).

**Table 4:** Schedule of the blood sample collection.

Sample Collection	Time of Blood Sample Collection *										
	Baseline	15 min	30 min	1 h	2 h	3 h	4 h	5 h	6 h	8 h	24 h
CBD and THC tests ^#^	X ^@^	X	X	X	X	X	X	X	X	X	X
Clinical lab tests ^&^	X										X

*—Blood samples were collected in EDTA vacutainers, processed, and plasma stored in −80 °C until transported to the Center for Human Toxicology for analysis. ^@^—time points when blood samples were taken. ^#^—Samples for CBD, THC, and their metabolite analysis. ^&^—Samples for clinical laboratory tests (Complete Blood Count (CBC) with platelet count and comprehensive metabolic panel.

## Data Availability

The original contributions presented in this study are included in the article/Supplementary Material. Further inquiries can be directed to the corresponding author.

## Supplementary Materials

The following supporting information can be downloaded at: https://www.mdpi.com/article/10.3390/ph19020231/s1, Figure S1: Individual plasma concentrations of CBD and 7-OH-CBD of participants who completed the study and were taking NHEC orally. A. Plasma concentrations of Cannabidiol (ng/mL); B. Plasma concentrations of 7-OH-Cannabidiol (ng/mL). HE-xx was used for de-identified designations of participants; Figure S2: Concentrations of CBD and 7-OH-CBD in the plasma of participants with normal BMI and overweight. A—Plasma concentrations of CBD (ng/mL). B—Plasma concentrations of 7-OH-CBD (ng/mL). Participants were grouped into participants with normal BMI (18.5–24.9) and overweight (25.0–30.0); Figure S3: Concentrations of CBD and 7-OH-CBD in plasma of females and males. A—Plasma concentrations of CBD (ng/mL). B—Plasma concentrations of 7-OH-CBD (ng/mL). Participants were grouped into females and males; Table S1: Urine THC test strip cross-reactivity.

## Abbreviations

The following abbreviations are used in this manuscript:

MCT	medium-chain triglyceride
BMI	body mass index
CTSI	Center for Clinical & Translational Science
CBC	Complete Blood Count
LC-MS/MS	liquid chromatography-tandem mass spectrometry
COOH-THC	carboxy delta-9-tetrahydrocannabinol
NHEC	natural hemp extract-based cosmetic
CBD	cannabidiol
THC	tetrahydrocannabinol
7-OH-CBD	7-hydroxy cannabidiol
Δ9-THC	delta-9 tetrahydrocannabinol
Δ8-THC	delta-8 tetrahydrocannabinol
11-OH-THC	delta-11 tetrahydrocannabinol
CBN	cannabinol
C_max_	maximum plasma concentration
T_max_	maximum plasma concentration
AUC_0–24_	area under the plasma concentration-time curve from zero to last measured concentration
AUC_0-inf_	AUC from time zero to infinity
T_1/2_	terminal (elimination) half-life
CL/F	apparent clearance
