# Supplementary material for: Results of an Exploratory Crossover Pharmacokinetic Study Evaluating a Natural Hemp Extract-Based Cosmetic Product: Comparison of Topical and Oral Routes of Administration
Source: Pharmaceuticals (Basel). 2026 Jan 29;19(2):231. doi: 10.3390/ph19020231 (PMC12943358; doi:10.3390/ph19020231)
Supplement: Supplementary file 1 [file pharmaceuticals-19-00231-s001.zip › pharmaceuticals-4051377-supplementary.pdf]

A. Plasma Concentrations of CBD

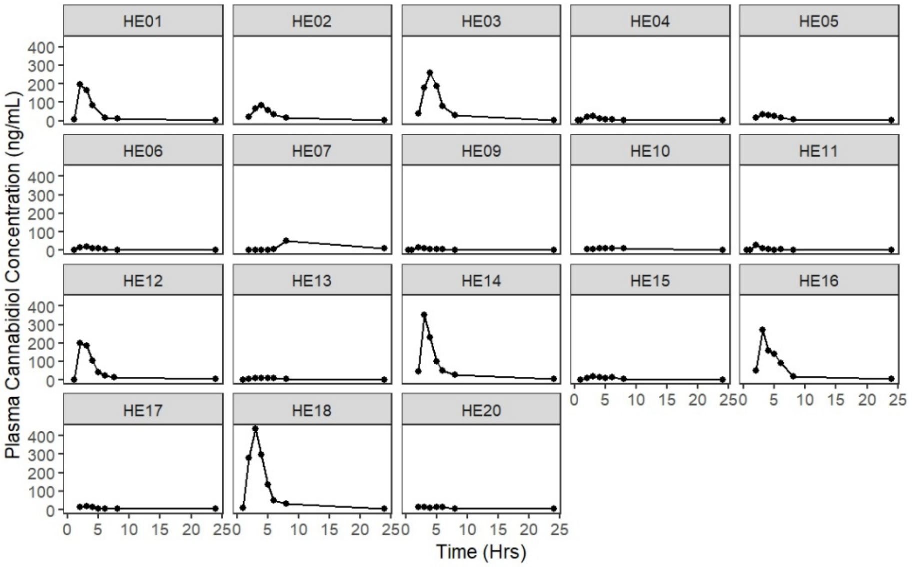

B. Plasma concentrations of 7-OH-CBD

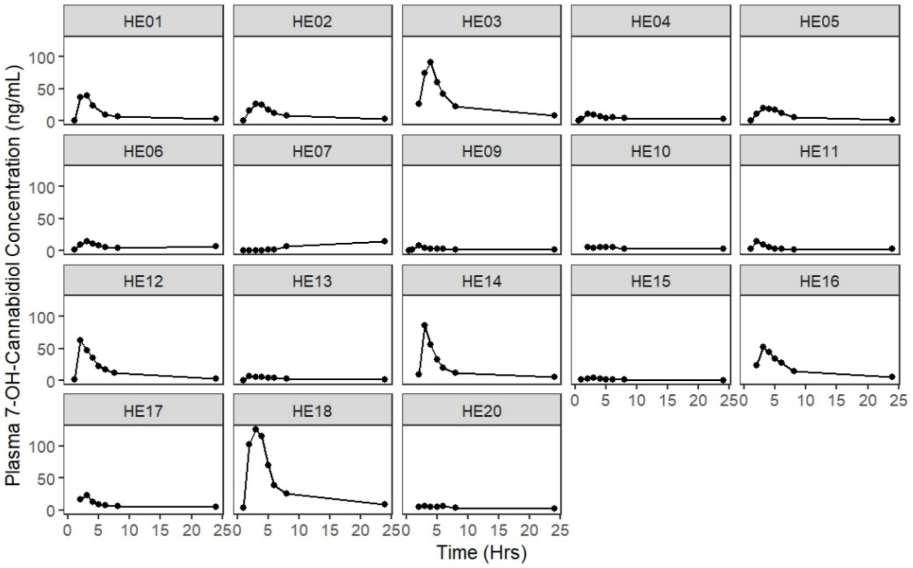

Figure S1. Individual plasma concentrations of CBD and 7-OH-CBD of participants who completed the study and were taking NHEC orally. A. Plasma concentrations of Cannabidiol (ng/ml); B. Plasma concentrations of 7-OH-Cannabidiol (ng/ml). HE-xx was used for deidentified designations of participants

A. Plasma CBD concentrations

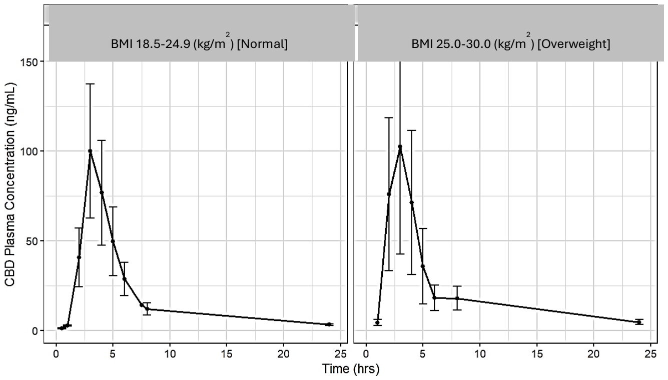

B. Plasma 7-OH-CBD concentrations

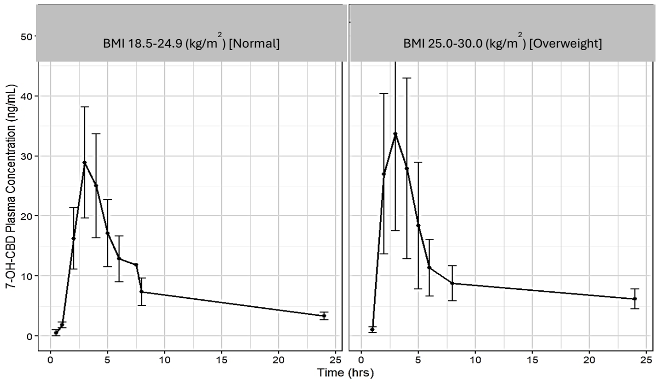

Figure S2. Concentrations of CBD and 7-OH-CBD in the plasma of participants with normal BMI and overweight. A - Plasma concentrations of CBD(ng/ml). B – Plasma concentrations of 7-OH-CBD (ng/ml). Participants were grouped into participants with normal BMI (18.5-24.9) and overweight (25.0-30.0)

A. Plasma CBD concentrations

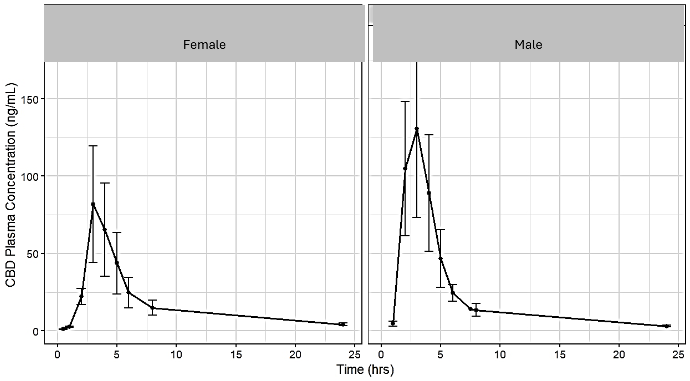

B. Plasma 7-OH-CBD concentrations

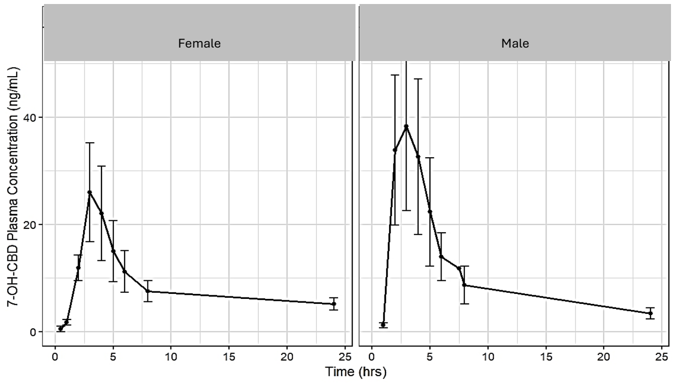

Figure S3. Concentrations of CBD and 7-OH-CBD in plasma of females and males. A - Plasma concentrations of CBD (ng/ml). B – Plasma concentrations of 7-OH-CBD (ng/ml). Participants were grouped into females and males

Table S1. Urine THC test strip cross-reactivity

Urine	Rest Replicates	Control, No CBD&THC	Low CBD, 50ng/ml	High CBD, 500ng/ml	Low THC, 50ng/ml	High THC 500ng/ml	Low THC+ High CBD
	1	negative	negative	negative	negative	positive	*negative*
**Lot 1**	2	negative	negative	negative	negative	positive	*negative*
	3	negative	negative	negative	negative	positive	*negative*
	1	negative	negative	negative	positive	positive	*negative*
**Lot 2**	2	negative	negative	negative	positive	positive	*negative*
	3	negative	negative	negative	positive	positive	*negative*
	1	negative	negative	negative	*negative*	positive	*negative*
**Lot 3**	2	negative	negative	negative	positive	positive	*negative*
	3	negative	negative	negative	positive	positive	*negative*
